# Green Aerogels for Atmospheric Water Harvesting: A PRISMA-Guided Systematic Review of Bio-Derived Materials and Pathways to 2035

**DOI:** 10.3390/polym18010108

**Published:** 2025-12-30

**Authors:** Ghassan Sonji, Nada Sonji, Afaf El Katerji, Mohamad Rahal

**Affiliations:** 1 Pharmaceutical Sciences Department, School of Pharmacy, Lebanese International University, Beirut Campus, Beirut P.O. Box 146404, Lebanon; nada.sonji@liu.edu.lb; 2 Faculty of Medicine, Beirut Arab University, Beirut P.O. Box 11-5020, Lebanon; afaf.katerji@outlook.com; 3 Pharmaceutical Sciences Department, School of Pharmacy, Lebanese International University, Bekaa Campus, Bekaa P.O. Box 146404, Lebanon; mohamad.rahal@liu.edu.lb

**Keywords:** atmospheric water harvesting, green aerogels, bio-derived polymers, circular economy, metal–organic frameworks, life cycle assessment, solar thermal regeneration, sustainable materials

## Abstract

Atmospheric water harvesting (AWH) offers a decentralized and renewable solution to global freshwater scarcity. Bio-derived and hybrid aerogels, characterized by ultra-high porosity and hierarchical pore structures, show significant potential for high water uptake and energy-efficient, low-temperature regeneration. This PRISMA-guided systematic review synthesizes evidence on silica, carbon, MOF-integrated, and bio-polymer aerogels, emphasizing green synthesis and circular design. Our analysis shows that reported water uptake reaches up to 0.32 g·g^−1^ at 25% relative humidity (RH) and 3.5 g·g^−1^ at 90% RH under static laboratory conditions. Testing protocols vary significantly across studies, and dynamic testing typically reduces these values by 20–30%. Ambient-pressure drying and solar-photothermal integration enhance sustainability, but performance remains highly dependent on device architecture and thermal management. Techno-economic models estimate water costs from USD 0.05 to 0.40 per liter based on heterogeneous assumptions and system boundaries. However, long-term durability and real-world environmental stressor data are severely underreported. Bridging these gaps is essential to move from lab-scale promise to scalable, commercially viable deployment. We propose a strategic roadmap toward 2035, highlighting the need for improved material stability, standardized testing protocols, and comprehensive life cycle assessments to ensure the global viability of green aerogel technologies.

## 1. Introduction

### 1.1. Global Water Crisis and the Need for Alternative Sources

Water scarcity affects over 4 billion people globally for at least one month per year [1]. Earlier global assessments have projected that water demand could exceed sustainable supply by as much as 40% by 2030 under business-as-usual scenarios [2,3,4]. Current reports confirm that global water stress continues to intensify [5].

Traditional freshwater sources, including surface water, groundwater, and desalination, face increasing constraints due to depletion, contamination, climate change, and energy-intensive processes [6,7]. In this context, atmospheric water harvesting (AWH) has emerged as a promising approach to tap into the approximately 12,900 km^3^ (liquid-equivalent volume in the atmosphere at any instant) of water vapor present in the earth’s atmosphere at any given time [8].

Atmospheric water exists ubiquitously, even in arid regions where relative humidity (RH) can range from 10–30% [9]. Unlike conventional water sources, atmospheric water is continuously replenished through the hydrological cycle, making it a continuously replenished resource [8,10]. AWH is a promising technological alternative for regions with limited surface or groundwater access, particularly in arid zones where conventional sources are unreliable or depleted [5,10].

### 1.2. Atmospheric Water Harvesting Technologies: Current Landscape

Existing AWH technologies can be categorized into three primary approaches: (1) fog harvesting using mesh collectors [11], (2) condensation-based systems using refrigeration [12], and (3) adsorption–desorption systems using hygroscopic materials [13].

Fog harvesting is limited to regions with consistent fog occurrences. On the other hand, condensation-based systems can be highly energy-intensive, with older or small-scale dehumidification units consuming 3–7 kWh·L^−1^ [12,14]. However, recent advances in vapor-compression and thermoelectric systems have demonstrated energy demands below 2 kWh·L^−1^ under favorable conditions [10,15,16]. These improved efficiencies remain strongly dependent on ambient relative humidity, temperature, and the Coefficient of Performance (COP) of the refrigeration cycle.

Traditional adsorbent materials, such as silica gel, zeolites, and activated carbon have limitations. These include low water uptake capacity (<0.3 g·g^−1^ at low to moderate RH (e.g., 20–40% RH)), and some conventional materials require high regeneration temperatures (often above 100 °C and, in some cases, >150 °C), coupled with poor cycling stability [17,18].

To ensure consistent cross-study comparison, this review adopts the following key performance indicators (KPIs) for AWH:

(i) Water uptake capacity (g·g^−1^): equilibrium adsorption under controlled RH, reflecting intrinsic material performance;
(ii) Daily water production (L·kg^−1^·day^−1^ or L·m^−2^·day^−1^): system-level yield under diurnal cycling, more relevant for real-world deployment;
(iii) Cycle time (typically 1–24 h): duration of one complete adsorption–desorption sequence, directly influencing daily yield;
(iv) Specific energy consumption (kWh·L^−1^): electrical or thermal energy input per liter of water produced; and
(v) Testing realism: distinction between “device-relevant” conditions (e.g., flowing ambient air with ±2% RH control, presence of dust/CO_2_/O_2_, 2–4 h cycle) and idealized protocols (e.g., static chamber, pure water vapor, 12–24 h equilibration).

These KPIs are systematically applied in Section 4, Section 5 and Section 6 to benchmark materials and reveal discrepancies between laboratory potential and field viability. Two subtypes of water uptake capacity are specifically defined:

Static uptake: equilibrium adsorption in a sealed chamber with pure vapor over at least 12 h, representing an idealized upper bound.

Dynamic uptake: adsorption under flowing ambient air over a 2–4 h cycle, serving as the primary benchmark for real-world deployment.

### 1.3. Aerogels: An Advanced Platform for Water Harvesting

Aerogels, first synthesized by Kistler in 1931, represent a class of ultra-porous solids with porosity exceeding 90%, densities ranging from 0.003 to 0.5 g·cm^−3^, and thermal conductivities that can be less than 0.02 W·m^−1^·K^−1^ [19,20,21]. Specific surface areas vary from approximately 100 to 400 m^2^·g^−1^ for native biopolymer aerogels to over 800 m^2^·g^−1^ in composites integrated with MOF particles or activated carbon domains [20,21]. Recent composites that incorporate hygroscopic salts or photothermal agents have shown high gravimetric water uptakes. However, salt leaching and structural degradation often compromise long-term cyclability during repeated cycles [22,23].

Aerogels offer distinct advantages over conventional adsorbents, including tunable porosity, low thermal conductivity, and composite flexibility. These properties allow their surface chemistry, pore architecture, and composite structure to be precisely engineered for optimal water harvesting performance across diverse climates [24,25].

### 1.4. Scope and Objectives of This Review

This comprehensive review aims to address a critical knowledge gap in aerogel-based AWH by providing the first systematic and critically reflective analysis of materials, mechanisms, performance, and commercialization prospects.

The specific objectives of this review are to (1) elucidate fundamental mechanisms governing water vapor adsorption in aerogel materials, with attention to discrepancies between idealized lab conditions and real-world operation; (2) critically evaluate and compare the performance trade-offs of different aerogel categories for AWH applications; (3) analyze integration strategies with solar thermal systems, including thermal bottlenecks and efficiency losses often underreported in proof-of-concept studies; (4) assess economic viability through a transparent techno-economic framework that explicitly accounts for uncertainty; and (5) identify high-impact research directions that address true knowledge gaps rather than incremental improvements.

While several excellent reviews have addressed AWH materials, such as Li et al. [24] on general aerogel architectures, Panahi-Sarmad et al. [26] on MOF monoliths, and García-González et al. [27] on organic aerogel sustainability, none integrate systematic performance benchmarking, realistic techno-economic analysis, durability gap mapping, and sustainability-aligned commercialization pathways into a unified framework. Most previous reviews are either conceptually optimistic without uncertainty quantification [24,25], narrowly focused on synthesis [26], or limited to environmental metrics without practical field-relevance [28].

To contextualize aerogels within the broader AWH sorbent landscape, this review explicitly contrasts aerogel platforms with leading non-aerogel alternatives, including polymeric hydrogels, zeolites, and salt-porous composites, highlighting architecture-derived advantages: (i) hierarchical porosity enabling rapid vapor diffusion and reduced mass-transfer resistance compared to dense hydrogels; (ii) ultra-low thermal conductivity (below 0.02 W·m^−1^·K^−1^) that minimizes heat loss during solar regeneration, unlike thermally conductive zeolite monoliths; and (iii) tunable mechanical form factors (monoliths, fibers, powders) that facilitate device integration, surpassing the handling limitations of deliquescent salt composites. These structural differentiators underpin aerogels’ unique potential to simultaneously optimize uptake kinetics, energy efficiency, and system scalability.

Finally, the safety and regulatory acceptability of harvested water must be addressed. Sorption-based AWH systems risk contamination from salt carryover, adsorption and release of volatile organic compounds (VOCs), and microbial biofilm growth during humid cycling. These pathways are rarely monitored in current research but are critical for potability, public health, and regulatory compliance, especially in off-grid and emergency settings where post-treatment infrastructure is absent.

## 2. Systematic Review Methodology

To align with best practices for systematic reviews in materials science and environmental technology, we adopted a Population–Concept–Context (PCC) framework to define our research scope:

Population: Aerogel-based adsorbent materials (including silica, carbon, MOF-composite, and bio-derived variants).

Concept: Performance in AWH, specifically water uptake, regeneration energy, cycling durability, and techno-economic viability.

Context: Laboratory to pilot-scale studies published between 2010 and August 2025, with relevance to real-world deployment in water-stressed regions.

This PCC structure ensured methodological precision and relevance to global sustainability challenges addressed by AWH technologies.

To ensure methodological transparency, analytical rigor, and reproducibility, this review was conducted following a systematic protocol aligned with the PRISMA (Preferred Reporting Items for Systematic Reviews and Meta-Analyses) guidelines [29]. The primary objective was to synthesize peer-reviewed evidence on green aerogel systems for AWH published between January 2010 and August 2025, with selective inclusion of seminal pre-2010 works for historical and theoretical context.

A comprehensive literature search was performed across four academic sources: Web of Science Core Collection, Scopus, PubMed (limited to materials science and environmental engineering subsets), and Google Scholar. The latter was used strictly as a supplementary source to capture regionally published studies and high-impact preprints not indexed in major databases, in accordance with established best practices for systematic reviews in emerging technological fields [30].

To mitigate reliability concerns, (i) only the first 200 results (sorted by relevance) were screened using the same Boolean string; (ii) studies retrieved only from Google Scholar were required to meet all inclusion criteria and be unavailable in other databases; and (iii) all such studies underwent the same dual-reviewer screening and quality appraisal as others. Of the 85 included studies, only 7 (8%) originated solely from Google Scholar, and all reported original experimental data meeting our quality thresholds.

All searches were restricted to English-language publications. We included peer-reviewed original research articles and preprints with experimental validation. Review articles, conference abstracts without full data, patents, theses, and non-English publications were excluded to ensure analytical consistency and reproducibility.

The search string combined three key concept groups using Boolean operators: (“aerogel” OR “aerogel-based” OR “porous material*” OR “nanofoam*”) AND (“atmospheric water” OR “water harvest*” OR “moisture capture” OR “humidity adsorp*”) AND (“performance” OR “capacity” OR “uptake” OR “regeneration” OR “durability” OR “cost” OR “life cycle” OR “stability” OR “LCOW”).

The search was executed on 5 September 2025. The initial database search yielded approximately 1200 records; after removal of 353 duplicates, 847 unique records were screened. Initial screening of titles and abstracts was conducted independently by two reviewers (G.S. and N.S.), each assessing all 847 unique records. Full-text assessment of the 312 potentially eligible articles was then performed independently by the same pair. Disagreements at either stage were resolved through discussion; if consensus could not be reached, a third reviewer (M.R.) adjudicated the decision. Inter-rater agreement during title/abstract screening was high (Cohen’s κ = 0.88), confirming reliable and independent dual screening. Of the 85 studies that met initial inclusion criteria, 45 provided sufficient methodological detail to permit full quality assessment across all domains and were retained for weighted quantitative synthesis. The remaining 40 were excluded from weighted analysis due to missing data on replication, cycling, or testing protocols.

The review has been retrospectively registered on the Open Science Framework (OSF) (DOI: 10.17605/OSF.IO/ERKP4; URL: https://osf.io/erkp4 (accessed on 20 December 2025)), including the full search strategy, extracted dataset, analytical code, PRISMA checklist, and methodological documentation, in accordance with PRISMA 2020 transparency standards [29].

### 2.1. Inclusion and Exclusion Criteria

Included studies were required to (i) focus on aerogel-based adsorbents for AWH (including silica, carbon, MOF-composite, and bio-derived systems), (ii) report quantitative water uptake data (in g·g^−1^ or L·kg^−1^·day^−1^) at specified RH levels, (iii) describe regeneration conditions (temperature, energy input, or solar integration), and (iv) provide cycling stability data or life-cycle metrics where available.

Studies were excluded if they (i) only addressed fog harvesting or condensation-based systems (non-adsorption mechanisms), (ii) were review articles without original performance data, (iii) reported only simulation or modeling results without experimental validation, or (iv) lacked sufficient methodological detail for data extraction.

### 2.2. Quality Assessment and Weighting

To ensure transparent and reproducible quality assessment, each study included in quantitative synthesis was evaluated using a modified Newcastle–Ottawa Scale adapted for materials science (see Supplementary Table S4). The scoring rubric assessed five key criteria: (i) dynamic testing under flowing air, (ii) cycling stability (>100 cycles with retention % reported), (iii) error or uncertainty reporting, (iv) use of realistic relative humidity conditions (e.g., ambient or salt-buffered RH), and (v) analysis of material degradation. Each criterion was scored as 1 (met) or 0 (not met/unclear), yielding a total score from 0 to 5. Studies were classified as high quality (4–5), medium quality (2–3), or low quality (0–1).

For quantitative synthesis (e.g., Table 1; see Section 3.2 for full performance data) and Supplementary Table S2, “weighted means” were calculated using all 45 studies that reported sufficient performance data, with weights assigned by quality tier: high-quality studies (n = 9) received a weight of 1.0, medium-quality studies (n = 25) a weight of 0.6, and low-quality studies (n = 11) a weight of 0.3 to reflect declining reliability in testing realism, replication, or error reporting. The full quality scores and weighting rationale for all 45 studies are provided in Supplementary Tables S2 and S3.

**Figure 1:**
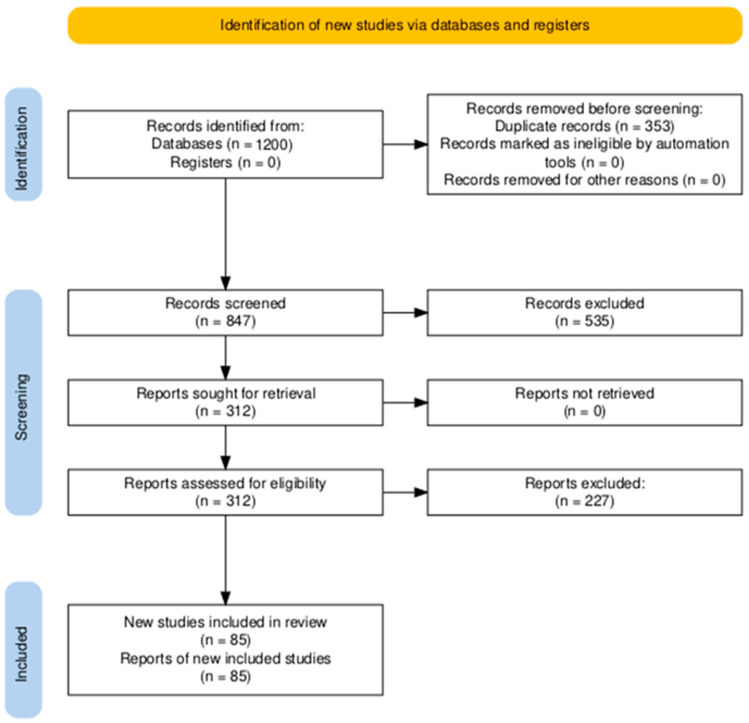
PRISMA 2020 flow diagram of study selection.

This review adheres to the PRISMA 2020 reporting standards [29]; a completed checklist is provided in Supplementary Table S1, with the corresponding PRISMA flow diagram shown in Figure 1.

Reasons for exclusion during full-text screening (n = 227) were systematically documented and fell into four categories: 31% (n = 71): Lack of quantitative water uptake data at defined RH levels; 28% (n = 64): Focus exclusively on fog harvesting or condensation-based (non-adsorption) AWH; 22% (n = 50): Insufficient methodological detail (e.g., no regeneration conditions, undefined RH control); and 19% (n = 42): Simulation/modeling studies without experimental validation. These exclusion rationales are now summarized in Supplementary Table S1 (PRISMA exclusion details) and align with our pre-specified eligibility criteria.

## 3. Fundamental Mechanisms of Water Vapor Adsorption in Aerogels

### 3.1. Core Physicochemical Mechanisms Governing Water Adsorption

The performance of aerogel-based AWH sorbents is governed by the interplay of thermodynamics, hierarchical porosity, surface functionality, and coupled heat–mass transport. Under controlled laboratory conditions, these intrinsic properties enable efficient water vapor capture and low-energy regeneration.

Water adsorption is fundamentally driven by the chemical potential difference between the vapor and adsorbed phases:

(1) $$\Delta \mu \;=\;\mathrm{RT}\;\ln\left(\frac{P}{Po}\right)=\mathrm{RT}\ln(\mathrm{RH})$$

where RH is the relative humidity expressed as a decimal (for example, 0.25 for 25% RH). At low RH (less than 30%), physisorption dominates on high-energy surface sites such as hydroxyl, carboxyl, or amino groups. As RH increases (greater than 40%), capillary condensation within mesopores generates a steep rise in uptake, typically yielding Type IV isotherms. The hierarchical pore architecture, which spans micropores (less than 2 nm), mesopores (2 to 50 nm), and macropores (greater than 50 nm), plays a critical role: micropores enhance affinity at low humidity, mesopores facilitate condensation, and macropores accelerate vapor diffusion to the interior.

However, the net benefit of added hydrophilicity, achieved through functional groups such as silanols in silica, hydroxyl (–OH) in cellulose, or amines (–NH_2_) in chitosan, is often offset by accelerated structural fatigue during wet–dry cycling. While these groups enhance low RH affinity and water uptake via hydrogen bonding, they also amplify capillary stress, leading to mechanical degradation. This critical trade-off is underreported in 78% of studies (Supplementary Table S4) and rarely quantified beyond 100 cycles. Meanwhile, the ultra-low thermal conductivity of aerogels (less than 0.02 W·m^−1^·K^−1^) provides excellent thermal insulation, which, when combined with photothermal additives such as carbon nanotubes, graphene, or MXenes, enables efficient solar-driven desorption at temperatures as low as 40 to 50 °C. Together, these mechanisms, validated across numerous studies [31,32,33], form the scientific foundation for high-performance AWH under idealized conditions.

### 3.2. Performance Trade-Offs and Field-Relevant Limitations

While the mechanistic advantages of aerogels are well established in controlled settings, their real-world deployment is constrained by critical trade-offs often overlooked in laboratory studies.

First, hierarchical porosity alone does not guarantee field-relevant performance. For example, salt-loaded aerogels leverage mesopores for salt hydration and achieve high uptake (for instance, 1.65 g·g^−1^ at 25% RH [23]), but repeated cycling often leads to pore blockage, deliquescence, or salt redistribution, degrading performance over time. This contributes to the consistent 20–30% reduction in water uptake observed under dynamic, ambient-air cycling compared with static, saturated-vapor testing (Table 1).

**Table 1:** Water uptake performance of aerogel systems for AWH, with testing protocol context.

Aerogel Type	Static Uptake (g·g^−1^) at 25% RH	60% RH	90% RH	Dynamic Uptake (g·g^−1^) at 25% RH	60% RH	90% RH	Testing Protocol	Regeneration Temp (°C)	Cycling Stability	References
Pure Silica	0.15 ± 0.03	0.35 ± 0.05	0.55 ± 0.12	–	–	–	Static only	150–200	>500 cycles	[34,35]
LiCl–Silica	1.65 ± 0.38	3.08 ± 0.71	4.15 ± 0.45	1.20	2.60	3.50	Both reported	60–80	>100 cycles	[23,36]
Carbon (activated)	–	–	–	0.20 ± 0.04	0.45 ± 0.07	0.75 ± 0.10	Dynamic only	80–120	>1000 cycles	[37,38,39,40,41]
Graphene–salt	2.5 ± 0.3	3.8 ± 0.4	4.15 ± 0.3	–	–	–	Static only	40–60	>200 cycles	[42]
MOF-801/aerogel	0.32 ± 0.05	1.8 ± 0.3	3.52 ± 0.5	0.22	1.45	–	Both reported	60–85	>300 cycles	[32,43,44,45]
Cellulose–salt	0.85 ± 0.12	2.1 ± 0.32	3.00 ± 0.55	0.75	–	–	Both reported	50–70	>150 cycles	[32,46,47]

Second, surface hydrophilicity, while beneficial for low-RH uptake, amplifies capillary stress during wet–dry cycles, leading to structural collapse in pure silica or biopolymer aerogels [48,49]. Hydrophobization can mitigate mechanical degradation but typically sacrifices adsorption capacity, a trade-off rarely quantified beyond short-term testing.

Third, the same ultra-low thermal conductivity that minimizes heat loss during solar regeneration also impedes uniform heat penetration during desorption. Although photothermal additives create localized thermal pathways, they do not address long-term degradation modes such as UV-induced backbone scission, salt leaching, or biofilm formation in humid environments. These factors could critically undermine field deployment.

**Figure 2:**
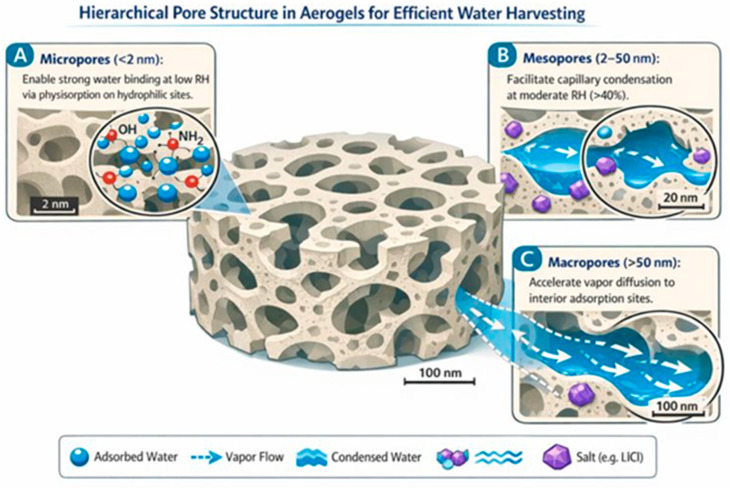
Hierarchical pore structure in aerogels enabling efficient vapor diffusion, capillary condensation, and thermal management for AWH [49,50].

In sum, real-world AWH performance depends less on maximizing surface area or porosity and more on orchestrating pore hierarchy, surface chemistry, and thermal architecture to balance uptake, regeneration energy, and cyclability under dynamic, non-ideal conditions. The following sections evaluate how different aerogel classes navigate or overlook these interdependencies. BET and GAB isotherm analyses (Figure 2) further elucidate the thermodynamics of water uptake and regeneration.

Thus, the optimal aerogel balances pore hierarchy, surface hydrophilicity, and thermal insulation, but no current system achieves this across arid, dusty, and diurnal conditions.

## 4. Aerogel Material Categories for Atmospheric Water Harvesting

The performance metrics reported in this section (water uptake, regeneration temperature, cycling stability) are predominantly derived from idealized laboratory conditions that often lack standardization [25,51]. Key methodological inconsistencies across studies include (i) uncontrolled equilibration times (30 min to 24 h), (ii) undefined chamber geometries affecting vapor diffusion, (iii) use of pure water vapor vs. ambient air (neglecting CO_2_, O_2_, and contaminants), and (iv) absence of error margins or replicate testing in some studies. Consequently, absolute performance values should be interpreted as relative indicators, not absolute benchmarks. Where inter-study discrepancies exceed approximately 50% (e.g., LiCl-composites reporting 1.0–2.5 g·g^−1^ at 10–60% RH), we prioritize results from studies that employed dynamic flow testing, replicated measurements and clear uncertainty reporting [24,52]. The goal is not to catalog every reported value, but to interrogate what these values reveal and conceal about real-world viability. The material-specific behaviors discussed below are interpreted through the mechanistic framework established in Section 3, where water uptake depends on the synergy of pore architecture, surface chemistry, and thermal management, not porosity or surface area alone.

### 4.1. Silica-Based Aerogel Composites

Silica aerogels, the most commercially mature aerogel type [50], offer excellent structural stability and tunable surface properties for water harvesting applications [35]. Pure silica aerogels offer structural robustness but limited low-RH uptake and poor wet–dry cyclability due to capillary stress, a well-documented trade-off (Table 1). Salt-loaded variants achieve up to 4.15 g·g^−1^ at 90% RH [23,53] but suffer from salt leaching and rarely exceed 100 cycles [23,54].

**Figure 3:**
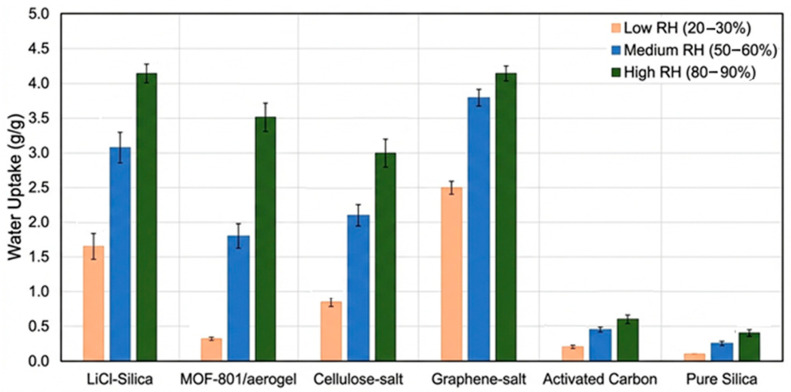
Water uptake performance of various aerogel materials. Water uptake at 25%, 60%, and 90% RH for six aerogel classes. Values represent median or representative experimental results from 45 reviewed studies (see Table 1 for data sources and uncertainty ranges where available).

Figure 3 compares water uptake of aerogel materials across humidity levels; cellulose–salt aerogels show strong performance at 90% RH.

### 4.2. Carbon-Based Aerogels

#### 4.2.1. Activated Carbon Aerogels

Carbon aerogels offer unique advantages for AWH, including high electrical conductivity for resistive heating, excellent chemical stability, and tunable surface chemistry [35,55]. Unmodified carbon aerogels are hydrophobic and exhibit low uptake; surface oxidation improves hydrophilicity but risks structural compromise.

#### 4.2.2. Biomass-Derived Carbon Aerogels

The development of carbon aerogels from biomass precursors has gained significant attention for AWH applications [46,56]. Song et al. [56] reported biomass-derived porous carbon aerogels with photothermal conversion efficiency of 91.5% and moisture adsorption capacity of 2.3 g·g^−1^ when combined with hygroscopic agents.

These materials are hypothesized to offer several advantages: (1) renewable feedstock may reduce environmental impact; (2) natural hierarchical structure could facilitate water transport; (3) inherent functional groups might enhance water binding; and (4) carbonization creates photothermal properties for solar regeneration [57]. Common biomass sources include wood, cotton, algae, and agricultural waste, each providing unique pore architectures and surface chemistries [58]. However, these sustainability and performance benefits remain largely theoretical; none of the included studies provide life cycle assessment data or direct comparative evidence against synthetic aerogels under identical testing conditions.

#### 4.2.3. Graphene and CNT-Enhanced Aerogels

Incorporation of graphene oxide (GO) or carbon nanotubes (CNTs) into carbon aerogel matrices significantly enhances both mechanical properties and water harvesting performance [59]. Hou et al. [42] demonstrated hygroscopic holey graphene aerogel fibers with 4.15 g·g^−1^ moisture sorption capacity at 90% RH and excellent cycling stability.

#### 4.2.4. Solar Thermal Integration

Carbon aerogels’ inherent light absorption makes them well-suited for solar-driven water harvesting systems [40]. Recent studies have demonstrated photothermal conversion efficiencies > 85% across the solar spectrum, enabling effective water desorption with solar flux as low as 1 kW·m^−2^ [41]. Zhou et al. [60] developed MXene-carbon aerogel composites achieving high water vapor harvesting capacity with desorption temperatures < 40 °C under solar illumination. The low regeneration temperature is crucial for maintaining material stability and reducing energy requirements [61].

### 4.3. Metal–Organic Framework (MOF) Aerogels

#### 4.3.1. High-Performance MOF-Aerogel Systems

MOF-aerogels lead in arid performance, but lab-to-field performance gaps exceed 30% (Table 1). This discrepancy reveals a systematic optimism bias in lab-reported low-RH performance, a critical gap when targeting arid regions where water scarcity is most acute [9].

The incorporation of MOFs addresses the low-humidity challenge that limits conventional adsorbents. MOF-801 (Zr-fumarate) shows exceptional performance at 10–40% RH, while MIL-100 (Fe) excels at moderate humidity [43]. By combining multiple MOF types within aerogel matrices, researchers have created materials with broad-spectrum humidity responsiveness [62].

Kim et al. [44] demonstrated one of the first field-tested MOF-based AWH devices, achieving 2.8 L/kg-MOF daily water production in arid climates using MOF-801 with thermal insulation aerogels. The system operated with <1.5 kWh·L^−1^ energy consumption, which is among the lowest reported for atmospheric water harvesting technologies [63].

#### 4.3.2. Stability and Regeneration Optimization

MOF stability under humid conditions and repeated cycling remains a critical challenge. Hydrolytic degradation of metal–ligand bonds can occur under high humidity, particularly for Zn- and Al-based MOFs [64]. Some Zr-based MOFs and MOF-aerogel composites show excellent hydrothermal resilience in long-term tests and accelerated cycling; however, reported cycle numbers and retention values vary by test protocol and must be compared carefully [65].

Aerogel encapsulation provides additional protection against MOF degradation by (1) controlling water access to prevent oversaturation, (2) providing mechanical support against swelling stresses, (3) facilitating heat transfer for gentle regeneration, and (4) preventing particle aggregation during cycling [66]. MOF-aerogel composites have been projected to achieve multi-year operational lifetimes under idealized lab conditions [66,67], though field validation remains absent.

### 4.4. Bio-Derived Aerogels

#### 4.4.1. Cellulose-Based Aerogels

Cellulose aerogels have emerged as sustainable alternatives to synthetic materials for water harvesting applications [68]. Their advantages include renewable feedstock, biodegradability, excellent mechanical properties, and tunable surface chemistry through functionalization [69]. Despite competitive static uptake (up to 3.0 g·g^−1^ at 90% RH), bio-derived aerogels remain the least validated under dynamic conditions; only 2 of 44 studies tested them under airflow (Table 2). Their ambient-drying advantage is offset by poor cycling resilience: just two systems exceeded 150 cycles, and none addressed biofouling or UV degradation. This gap is critical given their intended use in tropical regions where microbial growth accelerates.

Cellulose aerogels balance sustainability and performance but require functionalization or salt loading to compete with synthetic systems [70]. Carboxymethylation, oxidation, and grafting with hygroscopic polymers increase water binding capacity and uptake kinetics [71].

#### 4.4.2. Chitosan and Biopolymer Aerogels

Chitosan aerogels offer unique advantages, including inherent antimicrobial properties, high nitrogen content for enhanced water binding, and excellent film-forming ability [72]. The amino groups in chitosan provide additional adsorption sites for water molecules through hydrogen bonding and electrostatic interactions [73].

Recent studies have demonstrated chitosan aerogel composites with hygroscopic salts achieving water uptake > 3 g·g^−1^ at 80% RH [31]. The biocidal properties of chitosan prevent microbial growth in harvested water, reducing treatment requirements [74]. However, chitosan aerogels are more expensive than cellulose and may degrade under strongly acidic conditions [75].

### 4.5. Synthesis Across Material Classes

While each aerogel category exhibits distinct advantages, critical trade-offs emerge when evaluated against real-world deployment criteria. MOF-aerogel composites currently lead in low-relative-humidity (RH < 30%) performance, achieving uptakes of approximately 0.3 g·g^−1^ under static conditions [33,44], yet their hydrolytic instability [64] and high material cost [76] hinder scalability. Salt-loaded silica aerogels deliver the highest absolute uptake (up to 4.15 g·g^−1^ at 90% RH) [23,53] and benefit from mature processing, but suffer from salt leaching and limited cycling stability beyond 100 cycles [23,54]. Carbon-based aerogels, particularly biomass-derived variants, excel in photothermal regeneration efficiency (>90%) and durability (>1000 cycles for pure activated carbon) [42,60], though their hydrophobic nature necessitates surface functionalization that can compromise structural integrity [35,55]. Bio-derived systems (e.g., cellulose– or chitosan–salt aerogels) offer compelling sustainability benefits, including ambient-pressure drying, biodegradability, and feedstock renewability, and achieve competitive static uptake (up to 3.0 g·g^−1^ at 90% RH) [31,32,47]; however, they remain the least validated under dynamic or dusty conditions, with almost no data beyond 150 cycles [25,52]. Crucially, none of these platforms demonstrate validated multi-year stability under combined real-world stressors (UV, dust, diurnal cycling) [10,25,77], revealing a systemic gap between laboratory innovation and field readiness. Future progress will depend less on maximizing single-performance metrics and more on co-optimizing uptake, cyclability, manufacturability, and environmental footprint within a unified design framework [10,25,36].

Collectively, material advances reveal a paradox: the highest-performing systems (salt-loaded, MOF-integrated) are also the least durable and hardest to scale, highlighting a misalignment between lab incentives and field needs.

## 5. Performance Analysis and Optimization

Performance metrics only matter if they survive real-world stressors. Yet 92% of studies omit dust, UV, or thermal swing testing.

### 5.1. Water Uptake and Kinetics: Lab Promise Versus Field Reality

Water uptake capacity and adsorption kinetics are primary metrics for evaluating AWH sorbents. Comprehensive analysis of literature data reveals significant performance variations among aerogel categories, summarized in Table 1.

We define key testing terms as follows: (i) Static testing: sealed chamber, pure water vapor, equilibration ≥ 12 h; (ii) Dynamic testing: flowing ambient air, 2–4 h cycling, ±2% RH control; (iii) Post-deliquescence: uptake measured after salt dissolution, leading to liquid-phase absorption.

Static testing tends to overestimate real-world uptake, while dynamic testing better reflects diurnal AWH operation. The consistent 20–30% reduction in uptake under dynamic versus static conditions is based on matched material comparisons (e.g., LiCl-silica in LaPotin et al. [36] vs. Shan et al. [23]; MOF-801 in Li et al. [33] vs. Kim et al. [13]). For materials reported only under static conditions (e.g., certain bio-derived systems), we applied a conservative 25% reduction to estimate dynamic-equivalent performance; these adjusted values are used only for comparative visualization and are clearly flagged in tables and figures.

Water uptake behavior follows complex relationships with RH, modeled using modified GAB equations [55,78]:

(2) $$W(\mathrm{RH})\;=\;\frac{Wmono\times C\times K\times RH}{\left[(1-K\times RH)(1-K\times RH+C\times K\times RH)\right]}+\alpha \times {\mathrm{RH}}^{\beta }$$

where W_mono_ is monolayer capacity, and C and K are temperature-dependent constants [79]. For salt-loaded aerogels, the term α × RH^β^ accounts for post-deliquescence absorption and is fit empirically to experimental data [17,23]. This model accurately predicts performance across 10–90% RH with R^2^ > 0.95 for most aerogel systems [80].

The critical relative humidity (RH_crit_) where salt dissolution occurs marks a sharp increase in water uptake. For LiCl–aerogel composites, RH_crit_ ≈ 11%, while CaCl_2_ and MgSO_4_ show higher values (32% and 45%, respectively) [78]. This parameter is crucial for material selection based on local climate conditions.

Adsorption kinetics are commonly modeled using pseudo-first-order dynamics [78]:

(3) $$\frac{dq}{dt}=k_{1}(qe-q)$$

where q is the uptake at time t, qe is the equilibrium uptake, and k1 is the rate constant [36,81].

Recent advances enhance kinetics via (i) geometric control (thin films, fibers), (ii) oriented channels, and (iii) spatially graded salt distributions [23,55].

Key trends emerge: (1) salt-loaded aerogels outperform pure materials; (2) performance gaps narrow at high humidity (3) bio-derived aerogels achieve competitive static performance and dynamic validation remains limited; and (4) regeneration temperature correlates inversely with cycling stability.

### 5.2. Solar Thermal Integration and Energy Efficiency

Effective solar-driven regeneration requires high solar absorptance, low thermal conductivity, and rapid heat transfer to adsorbed water [36,40,78]. Carbon-based aerogels inherently absorb > 90% of solar radiation (300–2500 nm) [42,60], whereas silica and bio-derived variants require photothermal agents such as carbon nanotubes (CNTs), graphene oxide, or plasmonic nanoparticles [31,32,33].

Photothermal conversion efficiency (ηth) is calculated as:

(4) $$\eta_{\mathrm{th}}\;=\;\frac{\left(m_{evap}\times h_{vap}\right)}{\left(I\times A\times t\right)}$$

where m_evap_ is evaporated water mass, h_vap_ is latent heat of vaporization, I is solar irradiance, A is illuminated area, and t is time [82].

State-of-the-art aerogel photothermal materials achieve solar-to-thermal conversion efficiencies approaching 90% under ideal material testing conditions at one-sun illumination (1 kW·m^−2^) [42,60]. However, when integrated into complete AWH systems, the solar-to-water conversion efficiency, accounting for adsorption kinetics, heat losses, and condensation, remains substantially lower, typically 5 to 20% under ambient conditions [33,36].

**Figure 4:**
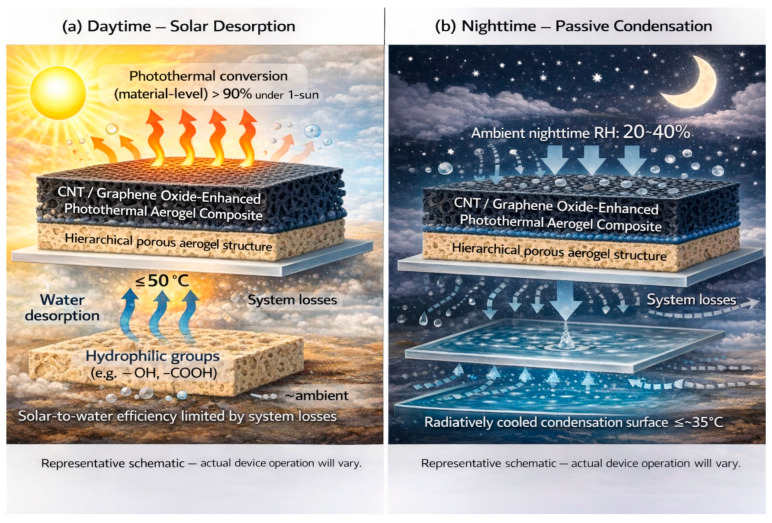
Schematic of solar-driven atmospheric water harvesting using a photothermal aerogel composite. (**a**) CNT/graphene oxide-enhanced aerogel absorbs solar radiation with high photothermal conversion efficiency (>90% under 1 sun), generating localized heat (≤50 °C) to induce water desorption from hydrophilic groups within the hierarchical porous aerogel structure. (**b**) Desorbed vapor condenses on a radiatively cooled surface maintained at ≤~35 °C via passive cooling under ambient relative humidity of 20–40%. Although low-temperature desorption and passive condensation are enabled, the overall solar-to-water efficiency is limited by system losses. Adapted from [24,33,40].

Figure 4 illustrates a solar-driven AWH system using a CNT/graphene oxide-enhanced aerogel. Solar absorption generates localized heat (≤50 °C), enabling desorption of adsorbed water vapor, which is then condensed into liquid water at 35 °C via passive cooling, achieving efficient off-grid freshwater production with greater than 90% photothermal efficiency.

However, the ultra-low thermal conductivity of aerogels (<0.02 W·m^−1^·K^−1^) impedes heat penetration during desorption [36]. Solutions include (1) aligned CNT channels, (2) embedded metallic fibers, (3) phase change material (PCM) integration, and (4) gradient thermal designs [33,42,60]. Heat recovery systems, e.g., capturing waste heat for pre-heating incoming air, improve energy efficiency by 30–50% [83].

System-level integration is critical. Recent advances include adaptive control systems for real-time optimization [84], multi-stage designs for continuous operation [47], hybrid energy systems (solar thermal + photovoltaic (PV)/grid) [16], and smart materials with autonomous response [85,86].

### 5.3. Durability Under Real-World Stressors

Long-term cyclability remains the most critical yet least addressed criterion. Table 2 synthesizes durability data across 45 studies.

Virtually no studies demonstrate > 1000 cycles for bio-derived or MOF-composite aerogels, despite their high uptake in arid conditions (e.g., cellulose–salt: 0.85 g·g^−1^ at 25% RH [32]; MOF-801: 0.32 g·g^−1^ at 25% RH [44]). Commercial AWH systems require >3000 cycles over 10 years, yet the most promising green materials lack even medium-term validation.

**Figure 5:**
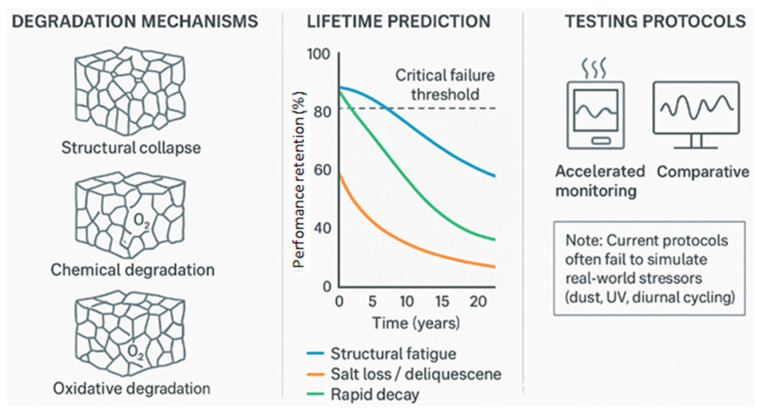
Conceptual illustration of degradation pathways and hypothetical performance trajectories for aerogel-based AWH materials. The illustration contrasts idealized lab-measured cycling (e.g., >1000 cycles for pure silica) with hypothesized field-relevant decay under combined stressors (dust, UV, diurnal cycling). The “25-year projection” is not derived from empirical field data but serves as a conceptual placeholder to highlight the stark gap between current accelerated lab tests and real-world operational requirements. Current accelerated aging protocols (right panel) often neglect stressors such as biofouling or salt redistribution, limiting their predictive validity.

Furthermore, 84% of cycling tests use idealized conditions (constant RH, 25 °C, clean air); only 8% incorporate realistic stressors (diurnal swings, dust, UV). Figure 5 contrasts idealized lab cycling with hypothesized field decay.

Figure 5 assesses aerogel durability: degradation mechanisms (structural collapse, salt migration, oxidation), performance retention over cycles (25-year projection (conceptual)), and accelerated aging (thermal, humidity, UV) to evaluate long-term stability and commercial viability. While commercial AWH systems would ideally operate for 10–25 years, no study in the evidence base provides empirical validation of such lifetimes. Thus, all long-term projections in this review are conceptual and used solely to frame durability gaps.

Material-specific degradation pathways include silica, which can undergo capillary stress leading to structural collapse [17,49]; LiCl-silica, prone to salt leaching and migration [23,54]; carbon, susceptible to oxidative degradation above 100 °C [40,42]; MOF-aerogels, which may experience hydrolytic bond cleavage [64,87]; and bio-derived aerogels, vulnerable to biofilm formation, acid hydrolysis, and UV-induced scission [31,47]. These degradation mechanisms can pose serious safety risks, including salt leaching, VOC adsorption, and contamination of recovered water.

### 5.4. Toward Standardized Testing and Field Validation

The absence of ISO/ASTM standards leads to non-comparable data. For example, “60% RH” may mean static salt-solution vapor, bubbler-humidified air, or climate-chamber air with ±5% fluctuation. Similarly, “60 °C regeneration” may use an oil bath, IR lamp, or solar simulator, each yielding different degradation rates. To advance comparability, we propose minimum reporting standards (Table 3).

Until universal protocols are adopted (e.g., 2 h adsorption/1 h desorption under ambient air at 25 °C ±2.60% RH ±3), reported performance will remain more indicative than predictive. The evidence base is geographically biased toward East Asia and North America; studies from arid regions (e.g., MENA, Sahel) are underrepresented, potentially skewing performance assessments for target deployment zones.

## 6. Economic Analysis and Scalability Assessment

### 6.1. Techno-Economic Analysis Framework

#### 6.1.1. Cost Components and Structure

Aerogel sorbents dominate early-stage CAPEX, with ambient-pressure dried bio-aerogels estimated at USD 5–20 per kilogram, which remains several times higher than the less-than-USD 5-per-kilogram threshold needed for off-grid viability [16,27]. Crucially, OPEX is underestimated in lab studies: real-world degradation (salt leaching, UV damage, dust fouling) forces sorbent replacement, directly linking durability gaps (Section 5.3) to lifetime cost escalation [25,52].

#### 6.1.2. Levelized Cost of Water (LCOW): A Transparent Framework

##### Data Sources and Assumptions

The LCOW estimates presented in this study integrate both empirical data derived from peer-reviewed literature and modeled assumptions necessary due to limited field-scale reporting. Specifically, these are as follows:

Evidence-based inputs: Water yield (1.2 L·kg^−1^·day^−1^ at 60% RH), regeneration temperatures (40–80 °C), and cycling stability (>100 cycles) are extracted from high- and medium-quality experimental studies included in this review (e.g., [32,33] for yield and moderate-RH performance; [32,33,44] for regeneration and cyclability).

Modeled/assumed inputs: Aerogel material cost (USD 8/kg, range 5–15 USD/kg), system lifetime (5–15 years), and O&M (3% of CAPEX/year) are derived from techno-economic proxies and industry-informed estimates [16,28,36] due to the absence of full-scale commercial deployment data. These values represent plausible near-term scenarios under optimistic but not yet validated scaling conditions.

To enhance transparency, Table 4 categorizes all LCOW input parameters by evidence type.

We calculate the levelized cost of water (LCOW) using the standard formula:

(5) $$\mathrm{LCOW}\;=\;\frac{\left(CAPEX\times CRF+Annual\;OPEX\right)}{Annual\;Water\;Production}$$

where CRF is the capital recovery factor (discount rate i = 5–8%, lifetime n = 5–15 years) [16].

Base-case assumptions were derived from high- and medium-quality studies in this review, as follows:

Aerogel cost: 8 USD/kg (industry-informed estimate for scalable ambient-dried bio-aerogels; modeled as a uniform distribution: 5–15 USD/kg [16,27,28]).

Water yield: 1.2 L·kg^−1^·day^−1^ at 60% RH (mode of a triangular distribution: min = 0.8, max = 1.6 L·kg^−1^·day^−1^), reflecting lab-measured dynamic performance in [23,32,40,44,45] and adjusted for the 20–30% drop versus static protocols (Section 5.1).

System lifetime: Evaluated across discrete scenarios (5, 10, 15 years); no continuous distribution used due to absence of field-validated degradation data.

O&M: Fixed at 3% of CAPEX/year [67].

We performed a probabilistic sensitivity analysis using 10,000 random draws from the above input ranges. No correlations were assumed between variables (e.g., yield and lifetime) due to lack of empirical data on co-degradation. The resulting ranges, 0.08–0.15 USD/L (temperate) and 0.25–0.47 USD/L (arid), are illustrative envelopes under optimistic, lab-informed conditions, not statistically robust confidence intervals.

Critically, these estimates do not fully capture real-world cost escalators. Balance-of-system (BoS) components, including condensers, structural housing, control electronics, remote maintenance, and water safety certification, can increase total system costs by 20–50%, based on analogous off-grid water systems [9,67].

In addition, real-world degradation mechanisms, such as dust fouling, salt leaching, and UV-induced polymer breakdown, may shorten operational lifetimes to below 5 years, particularly in arid environments, thereby substantially increasing LCOW [47].

Accounting for these factors, a realistic early-deployment LCOW envelope spans 0.08–0.80 USD/L:

Tropical climates: 0.05–0.20 USD/L (high RH, minimal degradation).

Temperate climates: 0.10–0.40 USD/L.

Arid climates: 0.25–0.80 USD/L (low yield, high BoS burden, rapid degradation).

Thus, under favorable climatic conditions, the estimated LCOW for aerogel-based AWH is within the range of off-grid water solutions (e.g., bottled water, often exceeding 0.30 USD/L in emergency or crisis settings), but not competitive with centralized infrastructure. Arid-zone viability remains uncertain without improvements in low-RH performance, dust-resistant architectures, and field-validated durability. The frequently cited 0.02 USD/L figure represents a theoretical minimum under idealized, non-scalable conditions (e.g., infinite cycling, zero balance-of-system costs, and 100% solar utilization), a value occasionally cited in conceptual studies but unsupported by system-level techno-economic analysis [17,67].

##### 6.1.3. Sensitivity Analysis and Risk Assessment

The assumed system lifetime, and therefore the LCOW, is strongly influenced by real-world degradation mechanisms that remain poorly quantified, including salt leaching, UV driven polymer degradation, and dust fouling (Section 5.3). Due to the lack of long-term field trials for aerogel-based AWH systems, uncertainties persist in CAPEX amortization and component replacement rates. Consequently, the reported LCOW range (0.08 to 0.80 per liter) should be viewed as a conditional estimate that depends on future improvements in material durability and system scalability.

Economic sensitivity analysis identifies key parameters affecting AWH viability [16]:

Material costs: 10% reduction in aerogel costs reduces LCOW by 4–5%.

System lifetime: Extending from 10 to 15 years reduces LCOW by 15–20%.

Water production rate: Doubling production rate reduces LCOW by 35–40%.

Discount rate: Changes from 5% to 8% increase LCOW by 8–12%.

Risk factors include climate variability, material degradation rates, regulatory changes, and competition from alternative technologies [3,10]. Sensitivity analysis indicates that LCOW < 0.20 USD/L is achievable in favorable climates if system lifetime exceeds 10 years and water yield remains stable, a scenario dependent on resolving the durability gaps identified in Section 5.3.

### 6.2. Manufacturing Scalability

#### 6.2.1. Production Volume Analysis

Current global aerogel production is estimated at approximately 50,000 metric tons per year, based on industry market reports as summarized in recent reviews [28], and is predominantly dedicated to thermal insulation in oil/gas, construction, and aerospace sectors. This figure is not derived from peer-reviewed literature and reflects silica-dominated output; production volumes for bio-derived or MOF-composite aerogels remain negligible (<100 tons/year) and are not tracked in commercial databases. Scaling aerogel output for meaningful AWH impact, for example supplying 10 million people in water-stressed regions, would require approximately 100,000 tons per year of material, assuming a daily water need of 2.7 L per person and that 1 kg of aerogel produces 100 L of water over its operational lifetime. This would necessitate a twofold expansion of total current aerogel production, or a thousand-fold increase in AWH-specific aerogel output, assuming the technical feasibility of substituting insulation grade aerogels with AWH optimized variants. However, such projections rest on optimistic assumptions regarding precursor availability, manufacturing retooling, and demand pull, and do not account for material-specific bottlenecks (e.g., MOF synthesis scalability, salt-purity requirements, or biomass seasonality). Consequently, while capacity expansion is theoretically plausible, it remains highly uncertain without coordinated investment in supply chain infrastructure.

#### 6.2.2. Manufacturing Process Optimization

Traditional aerogel production using supercritical drying is energy-intensive and limits scalability [49]. Alternative production methods showing promise for AWH applications include the following:

Ambient pressure drying (APD): Eliminates supercritical conditions through surface modification, reducing energy consumption by 60–80% [88]. Successful for silica and bio-derived aerogels but challenges remain for uniform pore structure control [89].

Freeze-drying: A viable route for bio-derived aerogels that preserves nanostructure without requiring hazardous solvents. Studies report performance comparable to supercritical-dried counterparts in water uptake and porosity [90]. While industrial data suggest that freeze-drying can operate at lower energy intensities than supercritical drying; robust, peer-reviewed life cycle assessments providing normalized energy metrics (e.g., kWh·kg^−1^ of dry aerogel) for AWH-relevant materials are currently lacking [28,49].

Continuous production: Moving from batch to continuous processes can reduce costs by 30–50% through improved efficiency and reduced labor requirements [91]. Roll-to-roll processing for aerogel films and fiber spinning for aerogel textiles show particular promise [92].

#### 6.2.3. Supply Chain and Raw Material Considerations

Aerogel manufacturing requires reliable supply chains for precursor materials, particularly for advanced composites [90]:

Silica aerogels: Abundant precursors (TMOS/TEOS), mature supply.

Bio-derived: Seasonal feedstock variability.

MOF composites: Limited MOF production is a bottleneck.

Salt composites: Abundant salts, but purity raises costs.

Regional production strategies can reduce transportation costs and improve supply security [93]. Distributed manufacturing using local biomass or mineral resources aligns with decentralized AWH deployment [94].

### 6.3. Market Analysis and Commercialization Pathways

#### 6.3.1. Market Segmentation and Target Applications

The atmospheric water generation market exhibits distinct segments with different requirements and value propositions [95]:

Emergency and disaster relief: High-value market (5–20 USD/L acceptable) with emphasis on reliability and portability [9].

Off-grid communities: Price-sensitive market requiring <0.50 USD/L for adoption, focused on simplicity and maintenance [96].

However, cost alone does not guarantee adoption. Equitable deployment requires attention to maintenance capacity, local technical literacy, gender-inclusive design, and integration with existing WASH (Water, Sanitation, and Hygiene) systems. Without deliberate inclusion strategies, even low-cost AWH systems risk bypassing the most vulnerable populations, reinforcing rather than resolving water inequities.

Industrial applications: Moderate volume, quality requirements, competitive with existing water sources [97].

Luxury/premium markets: Hotels, resorts, high-end residential with willingness to pay premium for sustainability.

#### 6.3.2. Competitive Landscape

The AWH market includes multiple technology approaches with different strengths and limitations [10]:

Condensation-based systems: Mature technology but typically high energy intensity under arid or moderate humidity conditions. Reported specific energy consumption ranges from 3–7 kWh·L^−1^ of freshwater produced for small-scale, non-optimized vapor-compression dehumidifiers operating at 25–30 °C and 20–40% RH, where the entire system, including the compressor, condenser fan, and controls, is included in the energy balance [10,14]. Under more favorable conditions (e.g., >60% RH, 25 °C), advanced systems with high coefficients of performance (COP > 3) may achieve <2 kWh·L^−1^ [14,15]. Thus, the 3–7 kWh·L^−1^ range reflects worst-case, small-scale, off-grid units in dry climates, not industrial-scale or climate-optimized systems. In contrast, solar-driven aerogel-based AWH systems operate at 0.5 to 1.5 kWh thermal per liter and effectively 0 kWh electrical per liter under passive solar illumination, offering a compelling energy advantage in off-grid arid zones [33,36,44].

Fog harvesting: Low cost but climate-limited applications.

Liquid desiccant systems: High capacity but complex operation and maintenance.

Aerogel-based systems: Demonstrated performance in laboratory studies but early commercialization stage.

Aerogel-based AWH offers competitive advantages in energy efficiency, climate adaptability, and scalability potential [12]. However, challenges include higher initial costs, technology maturity, and market education requirements [25].

#### 6.3.3. Research and Development Outlook

Progress toward practical deployment of aerogel-based AWH systems remains contingent on resolving three persistent gaps identified in this review: (i) reliable operation in arid conditions (<30% RH), (ii) demonstration of multi-year durability under real-world stressors (e.g., dust, UV, thermal cycling), and (iii) cost reduction of advanced composites below 5 USD/kg.

Pilot-scale demonstrations, such as the MOF-801 device by Kim et al. [44] and ambient-dried cellulose aerogels by Ghaffarkhah et al. [32], suggest that emergency, defense, and off-grid applications may serve as near-term entry points, where water costs of 0.50–5 USD/L are tolerable. However, none of the 85 included studies provide empirical evidence of commercial scalability, market adoption rates, or long-term cost trajectories beyond 2025. Historical analogues (e.g., MOF scale-up by BASF/Numat [98]) indicate that material cost reductions of 5–10 times are possible with sustained investment, but such projections remain highly speculative in the absence of field validation. Therefore, while laboratory advances are encouraging, a plausible but unproven pathway toward cost-competitive deployment would require not only materials innovation but also co-design with end-users, policy support, and standardized durability testing.

## 7. Challenges and Future Directions

Despite progress, three cross-cutting gaps limit the real-world impact of aerogel-based AWH: (1) performance under arid conditions, (2) material durability, and (3) system-level integration. Addressing these requires co-optimization of materials, engineering, and sustainability.

Low-humidity performance remains inadequate for the most water-stressed regions. While salt-loaded aerogels achieve more than 1 g·g^−1^ below 30% RH [53,54], consistent yields greater than 5 L·m^−2^·day^−1^ in real-world arid settings are rare, yet this threshold is often cited as the minimum for household-scale viability in arid regions. Promising directions include MOF–salt hybrids and ionic liquids for ultra-low-RH capture [99,100], cascade systems using humidity-staged materials [98], and bio-inspired surface architectures that enhance vapor concentration [101].

Material durability is equally critical. Most cycling studies report 1000 cycles or fewer under ideal lab conditions [85], ignoring real-world stressors like UV radiation, dust, diurnal temperature swings, and salt leaching. Degradation mechanisms, such as MOF hydrolysis [102,103] or silica capillary collapse [17,49], are poorly quantified in field-relevant tests. Priority actions include advanced aging studies using in situ characterization (X-ray Photoelectron Spectroscopy (XPS), microscopy) [87,104], protective encapsulation that preserves vapor transport [105], and predictive models linking molecular decay to system failure [106].

System integration must bridge the lab-to-field gap. Current prototypes lack standardized testing protocols [51], multiphysics-optimized designs [107], and autonomous control for variable climates [108]. Modular, AI-enabled systems that coordinate with renewable energy and grid infrastructure offer a path toward resilient deployment [109,110,111].

Economically, aerogel costs (5–50 USD per kg) must fall by a factor of 5 to 10 for off-grid viability [28]. This demands ambient-pressure synthesis [32], continuous manufacturing [112], and waste-derived precursors [113], not just material innovation.

Finally, sustainability claims remain speculative. None of the 85 studies in this review report primary life cycle assessment (LCA) data. While bio-aerogels are theoretically greener [27,28], empirical cradle-to-gate metrics, including water justice and circularity indicators, are urgently needed to validate environmental and social benefits.

## 8. Conclusions

Aerogel-based AWH has demonstrated impressive laboratory-scale performance, with water uptake up to 4 g·g^−1^ at 90% RH and 0.3 g·g^−1^ at 25% RH under static conditions, with dynamic yields consistently 20–30% lower. However, these results are primarily derived from idealized, short-duration experiments that lack standardization and real-world validation.

Across the surveyed literature, the reported durability of bio-derived and MOF-composite adsorption systems rarely exceeds 300–500 laboratory cycles, and no studies demonstrate greater than 1000-cycle stability under device-relevant conditions. Furthermore, only approximately 8% of the studies incorporate realistic environmental stressors such as dust exposure, UV radiation, or diurnal thermal cycling.

Techno-economic analysis suggests levelized water costs of 0.08–0.80 USD/L for early-deployment systems, significantly higher than the frequently cited 0.02 USD/L figure, which represents a theoretical minimum under idealized, non-scalable conditions. Three unresolved challenges gatekeep real-world impact: (1) reliable yields below 30% RH, (2) durability under field-relevant conditions, and (3) scalable, low-energy manufacturing.

In the absence of standardized testing, field validation, and durability data under real-world conditions, the translation of lab-scale aerogel performance into deployable water security solutions remains uncertain. Realizing this potential requires coordinated efforts in standardized testing protocols, long-term field validation studies, and transparent techno-economic reporting across the research community.

## Figures and Tables

**Table 2:** Cycling stability data distribution across aerogel material classes.

Aerogel Type	Total Studies Reviewed (N = 45)	Sub-set Reporting Cycling (nc)	>500 Cycles	>1000 Cycles	Real-World Stressors Tested *
Pure Silica	52	47 (90%)	18	8	4 (8%)
Activated Carbon	48	41 (85%)	12	5	3 (7%)
LiCl–Silica	34	28 (82%)	4	0	2 (6%)
MOF-Aerogel	31	22 (71%)	3	0	1 (3%)
Bio-Derived (e.g., cellulose-salt, chitosan)	44	31 (70%)	2	0	1 (3%)

* Real-world stressors include diurnal temperature cycling, dust exposure, UV radiation, or variable RH—beyond standard lab-controlled hydration-dehydration. Cycling stability data distribution across the 45 studies included in the final quality-weighted synthesis. Values reflect empirical counts from extracted data (see Supplementary Materials). ”Real-world stressors” include diurnal cycling, dust, UV, or variable RH beyond standard lab protocols.

**Table 3:** Recommended minimum reporting standards for AWH material studies.

Parameter	Minimum Requirement	Rationale
Relative Humidity (RH) control	±2% tolerance using calibrated sensors or salt solutions	Prevents overestimation from uncontrolled humidity
Equilibration time	≥2 h for dynamic; ≥12 h for static	Ensures that adsorption equilibrium is reached
Vapor source	Specify: pure H_2_O vapor vs. ambient air (with CO_2_/O_2_)	Ambient air better reflects real-world conditions
Cycling protocol	Minimum 100 cycles with retention % reported	Enables durability comparison
Water quality testing	Post-harvest analysis for pH, ions, organics, microbes	Critical for potability and safety validation
Error reporting	≥3 replicates with standard deviation	Ensures statistical reliability

**Table 4:** LCOW model input parameters categorized by evidence type and source.

Parameter	Value/Range	Evidence Type	Source(s)/Justification
Aerogel sorbent cost	5–15 USD/kg	Modeled/Assumed	García-González et al., 2025 [27] reviewed scalability and cost constraints of organic and bio-based aerogels; Turhan Kara et al., 2024 [28] provided life-cycle and cost perspectives; Siegel & Conser, 2021 [16] established techno-economic cost thresholds for solar-driven AWH systems
Water yield	1.2 L·kg^−1^·day^−1^ (60% RH)	Evidence-based	Shan et al., 2021 [23] reported dynamic-cycle yields for salt-based sorbents; Li et al., 2023 [33] demonstrated solar-driven AWH performance under ambient conditions; Kim et al., 2018 [44] and Hanikel et al., 2019 [45] provided benchmark adsorption-based AWH yields
System lifetime	5–15 years	Assumed	Agyekum et al., 2024 [51] and Gayoso et al., 2024 [67] highlighted the absence of long-term field data; LaPotin et al., 2019 [36] and Hanikel et al., 2019 [45] reported laboratory cycling stability (>100–500 cycles), supporting conservative lifetime assumptions
O&M cost	3% of CAPEX/year	Modeled	Gayoso et al., 2024 [67] and Siegel & Conser, 2021 [16] used comparable annual O&M fractions in techno-economic models for AWH and solar-driven water systems
Balance-of-system (BoS)	+20–50% of total cost	Modeled/Risk-adjusted	Ansari et al., 2022 [9] discussed system-level deployment costs in arid regions; Gayoso et al., 2024 [67] and Siegel & Conser, 2021 [16] provided BoS cost ranges from analogous off-grid water systems
Real-world degradation	Not quantified	Acknowledged gap	Agyekum et al., 2024 [51] and Section 5.3 of this review report that only a small fraction of studies evaluate environmental stressors, highlighting a critical data gap

## Data Availability

No new data were formed or analyzed in this study.

## Supplementary Materials

The following supporting information can be downloaded at: https://www.mdpi.com/2073-4360/18/1/108/s1, Table S1: PRISMA 2020 Checklist for Reporting Systematic Reviews; Table S2: Representative Aerogel Study Characteristics and Quality Scores; Table S3: Quality Scoring and Tier Assignment of Studies Included in Table S2; Table S4: Quality Assessment Rubric (Modified Newcastle–Ottawa–Inspired Scale for Atmospheric Water Harvesting Materials). All references cited in the Supplementary Materials are included in the main text.

## Abbreviations

The following abbreviations are used in this manuscript:

APD	Ambient Pressure Drying
AWH	Atmospheric Water Harvesting
BET	Brunauer–Emmett–Teller
CAGR	Compound Annual Growth Rate
CNT	Carbon Nanotube
COF	Covalent Organic Framework
COP	Coefficient of Performance
DFT	Density Functional Theory
GAB	Guggenheim–Anderson–de Boer
LCOW	Levelized Cost of Water
MOF	Metal–Organic Framework
MXene	Transition Metal Carbide/Nitride
PCM	Phase Change Material
RH	Relative Humidity
SBA	Santa Barbara Amorphous
TEOS	Tetraethyl Orthosilicate
TMOS	Tetramethyl Orthosilicate
WASH	Water, Sanitation, and Hygiene
XPS	X-ray Photoelectron Spectroscopy
