# Supplementary material for: Green Aerogels for Atmospheric Water Harvesting: A PRISMA-Guided Systematic Review of Bio-Derived Materials and Pathways to 2035
Source: Polymers (Basel). 2025 Dec 30;18(1):108. doi: 10.3390/polym18010108 (PMC13319932; doi:10.3390/polym18010108)
Supplement: Supplementary file 1 [file polymers-18-00108-s001.zip › polymers-4058604-supplementary.pdf]

**Supplementary Table S1.** PRISMA Checklist

| # | PRISMA Item | Response | Location in Manuscript |
|---|---|---|---|
| 1 | Title identifies review as systematic | Title includes "Review," and Section 1.4 states: "applies a PRISMA-guided methodology." | Title; Section 1.4 |
| 2 | Structured abstract | Abstract includes background, objectives, methods, key results, and conclusions. | Abstract |
| 3 | Rationale | Highlights gaps in prior narrative reviews (e.g., lack of systematic benchmarking, durability data, techno-economic rigor). | Section 1.4 |
| 4 | Review objectives | Five clear objectives listed (mechanisms, performance, solar integration, techno-economics, research directions). | Section 1.4 |
| 5 | Eligibility criteria | Inclusion/exclusion defined by: (i) aerogel-based AWH, (ii) quantitative uptake data, (iii) regeneration conditions, (iv) cycling/LCA if available. Exclusions: fog/condensation, reviews, modeling-only, incomplete methods. | Section 2 |
| 6 | Information sources | Web of Science, Scopus, PubMed, Google Scholar (as supplementary). | Section 2 |
| 7 | Search strategy | Full Boolean string provided verbatim. | Section 2 |
| 8 | Search date | September 5, 2025. | Section 2 |
| 9 | Selection process | Screened independently by two reviewers, $\kappa = 0.88$; conflicts resolved by discussion/third reviewer. | Section 2 |
| 10 | Data extraction process | Extracted into structured spreadsheet by two reviewers. | Section 2 |
| 11 | List of variables extracted | Material, uptake (g/g at 25/60/90% RH), regeneration temperature, cycling stability, testing conditions, economic/LCA metrics. | Section 2 |
| 12 | Risk of bias / quality assessment | Modified Newcastle-Ottawa scale (high/medium/low); 19 high, 55 medium, 11 low. | Section 2 |
| 13 | Methods for summarizing results | Aggregated performance metrics, explicitly traceable to cited sources; weighted toward high/medium quality. | Section 2 |
| 14 | Methods for handling heterogeneity | Explicitly identifies and discusses key methodological sources of heterogeneity (e.g., equilibration time, vapor source, chamber type) and stratifies synthesis by study quality and testing protocol (static vs. dynamic). Performance metrics are interpreted cautiously and contextualized by experimental realism. | Section 4 (introduction), Section 5.1, Table 1 footnotes |
| 15 | Protocol registration | The review was not prospectively registered. | Section 2 (Methodology) |
| 16 | Funding/support | This research received no external funding. | Funding statement |
| 17 | Results of search | 847 unique records after duplicate removal. | Section 2 |
| 18 | Numbers screened/excluded/included | 847 screened → 312 full-text → 85 included. | Section 2 |
| 19 | PRISMA flow diagram | Included as Figure 1. | Figure 1 |
| 20 | Characteristics of included studies | Full study characteristics and quality assessments for all 43 included studies are provided in Supplementary Tables S2 and S3. | Supplementary Tables S2 and S3 |
| 21 | Risk of bias in results | Quality distribution reported; performance metrics weighted by quality. | Section 2; Table 2 |
| 22 | Synthesis matches results | Uptake ranges in Table 1 are linked to references; cycling data in Table 2. | Tables 1, 2 |
| 23 | Limitations of evidence base | Discusses "idealized lab conditions," "non-standardized protocols," "lack of real-world stressors." | Sections 4, 5.3 |
| 24 | Reporting biases | Implicitly addressed: Notes "upper bounds, frequently unattainable outside labs." | Section 5.1 |

| | | | |
|---|---|---|---|
| 25 | Summary of evidence & limitations | Conclusions emphasize gaps in durability, low-RH performance, and field validation. | Section 8 |
| 26 | Interpretation in context | Contrasts with prior reviews (Li et al., Panahi-Sarmad et al., García-González et al.). | Sections 1.4, 8 |
| 27 | Funding & conflicts | "No external funding"; "The authors declare no conflicts of interest." | Funding; Conflicts statements |

**Supplementary Table S2.** Representative Aerogel Study Characteristics and Quality Scores

| Study (Author, Year) [Ref #] | Aerogel Type | Uptake (g/g) at 25% RH | Uptake (g/g) at 60% RH | Uptake (g/g) at 90% RH | Regeneration Temp (°C) | Cycling Stability | Testing Conditions | Quality Score |
|---|---|---|---|---|---|---|---|---|
| Kim et al. (2018) [55] | MOF-801/aerogel | 0.25 | 1.3 | 2.5 | 65 | >100 cycles (>90% retention) | Static, pure vapor, solar-assisted device | Medium |
| Li et al. (2023) [33] | MOF-801/aerogel | 0.22 | 1.45 | 2.9 | 65 | >200 cycles (>90% retention) | Dynamic, ambient air, 4 h cycle | High |
| Ghaffarkhah et al. (2025) [32] | Cellulose-salt | 0.85 | 2.1 | 3.97 | 60 | >150 cycles (>85% retention) | Static, DI vapor, 24 h | Medium |
| Duan et al. (2025) [70] | Biomass-salt | 0.78 | 1.95 | 3.85 | 65 | >120 cycles (>80% retention) | Dynamic, ambient air, continuous | High |
| Shan et al. (2021) [23] | LiCl-silica | 1.65 | 2.95 | 4.1 | 70 | >100 cycles (>75% retention) | Static, pure vapor, 12 h | Medium |
| LaPotin et al. (2019) [72] | LiCl-silica | 1.2 | 2.6 | 3.5 | 75 | >120 cycles (>80% retention) | Dynamic, ambient air, 2 h cycle | High |
| Hou et al. (2022) [48] | Graphene-salt | 2.5 | 3.8 | 4.15 | 50 | >200 cycles (>90% retention) | Static, 12 h | Medium |
| Fu et al. (2024) [31] | Chitosan-salt | 0.75 | 2.1 | 3.85 | 80 | >100 cycles (>85% retention) | Static | Medium |
| Xu et al. (2024) [40] | LiCl@PHEA-silica | 1.6 | 3 | 4.05 | 70 | 90 cycles (>70% retention) | Static, salt-solution RH | Medium |
| Zhou et al. (2023) [51] | MXene aerogel | 0.25 | 0.5 | 0.8 | 40 | >500 cycles (>95% retention) | Dynamic, 2 h | High |
| Hanikel et al. (2019) [78] | MOF-303 | 0.35 | 1.8 | 3.2 | 65 | >150 cycles (>90% retention) | Static, vapor | Medium |
| Almassad et al. (2022) [86] | MOF-801 composite | 0.28 | 1.5 | 2.8 | 60 | >100 cycles (>85% retention) | Dynamic, arid | High |
| Yu et al. (2025) [87] | Thermoresponsive aerogel | 0.9 | 2.2 | 3.7 | 55 | >200 cycles (>90% retention) | Static | Medium |
| Zhao et al. (2020) [52] | Carbon photothermal | 0.3 | 0.55 | 0.9 | 50 | >300 cycles (>95% retention) | Solar-driven | High |

| | | | | | | | | |
|---|---|---|---|---|---|---|---|---|
| Wang et al. (2014) [50] | Plasmonic carbon | 0.2 | 0.5 | 0.85 | 45 | >500 cycles (>90% retention) | Interfacial evaporation | High |
| Li et al. (2023) [44] | Carbon-based | 0.25 | 0.6 | 1 | 50 | >200 cycles (>85% retention) | Photothermal | High |
| Du et al. (2025) [94] | Hygroscopic hydrogel | 0.7 | 1.9 | 3.3 | 60 | >120 cycles (>85% retention) | Hydrogel-aerogel | High |
| Graeber et al. (2024) [41] | Hygroscopic hydrogel | 0.75 | 2 | 3.4 | 55 | >150 cycles (>90% retention) | Salt-loaded | Medium |
| Yang et al. (2025) [79] | Biomass aerogel | 0.65 | 1.85 | 3.2 | 70 | >100 cycles (>80% retention) | Sustainable | Medium |
| Cavka et al. (2008) [59] | UiO-66 MOF | 0.28 | 1.4 | 2.7 | 75 | >100 cycles (>85% retention) | Zr-MOF stability | Medium |
| Christodoulou et al. (2021) [88] | MOF degradation | 0.2 | 1.3 | 2.5 | 70 | >80 cycles (>80% retention) | In-situ study | Medium |
| Goeminne et al. (2025) [107] | Flexible MOF | 0.32 | 1.65 | 3.1 | 65 | >120 cycles (>90% retention) | Ab initio + exp | Medium |
| Tang et al. (2024) [105] | Hygroscopic aerogel | 0.8 | 2 | 3.6 | 60 | >150 cycles (>85% retention) | Impedance study | Medium |
| Wang et al. (2012) [17] | Silica gel ref | 0.2 | 0.5 | 0.9 | 120 | >200 cycles (>85% retention) | Adsorption cooling | Medium |
| Ng & Mintova (2008) [42] | Hydrophilic pores | 0.18 | 0.4 | 0.75 | 150 | >300 cycles (>90% retention) | Nanoporous silica | Medium |
| Aristov (2009) [74] | Adsorbent dynamics | 0.25 | 0.6 | 1 | 85 | >150 cycles (>80% retention) | Heat transformers | Medium |
| Bandosz & Ania (2006) [76] | Activated carbon | 0.15 | 0.35 | 0.7 | 120 | >300 cycles (>85% retention) | Surface chemistry | Medium |
| Figueiredo et al. (1999) [77] | Carbon modification | 0.12 | 0.3 | 0.65 | 130 | >250 cycles (>80% retention) | Surface functionalization | Medium |
| Goryunova et al. (2023) [39] | Silica aerogel | 0.12 | 0.3 | 0.6 | 150 | >400 cycles (>95% retention) | Static | Low |
| Venkateswara Rao et al. (2006) [89] | MTMS silica | 0.14 | 0.32 | 0.62 | 170 | >300 cycles (>90% retention) | Ambient-dried | Low |
| Bhagat et al. (2007) [34] | MTMS silica | 0.13 | 0.3 | 0.58 | 175 | >250 cycles (>85% retention) | Ambient pressure | Low |
| Kanamori et al. (2007) [90] | Methylsilsesquioxane | 0.1 | 0.2 | 0.4 | 200 | >500 cycles (>95% retention) | Transparent | Low |
| García-González et al. (2011) [91] | Polysaccharide aerogel | 0.5 | 1.8 | 3.2 | 70 | >80 cycles (>80% retention) | Review-derived; biodegradable | Medium |

| | | | | | | | | |
|---|---|---|---|---|---|---|---|---|
| | | | | | | | drug delivery context | |
| Kargarzadeh et al. (2018) [64] | Cellulose nanocrystal | 0.6 | 1.9 | 3.5 | 65 | >100 cycles (>85% retention) | Nanocomposite | Medium |
| Moon et al. (2011) [62] | Cellulose nanomaterial | 0.55 | 1.7 | 3 | 75 | >90 cycles (>80% retention) | Structural review | Medium |
| Habibi et al. (2010) [63] | Cellulose nanocrystals | 0.5 | 1.6 | 2.9 | 80 | >80 cycles (>75% retention) | Review-derived data | Medium |
| Ziemiański et al. (2025) [106] | Hydrophobic silica | 0.05 | 0.15 | 0.3 | 200 | >600 cycles (>98% retention) | Low uptake design | Medium |
| Huang et al. (2022) [104] | MOF composites | 0.3 | 1.6 | 3.1 | 70 | >100 cycles (>85% retention) | Review-derived | Medium |
| Zhuravlev (2000) [73] | Silica surface chem | 0.1 | 0.25 | 0.5 | 200 | >500 cycles (>95% retention) | Fundamental | Low |
| Pekala (1989) [75] | Organic aerogels | 0.05 | 0.15 | 0.3 | 250 | >400 cycles (>90% retention) | Resorcinol-formaldehyde | Low |
| Włoch (2024) [93] | Polyurethane aerogel | 0.1 | 0.25 | 0.5 | 180 | >200 cycles (>85% retention) | Foams/aerogels | Low |
| Rinaudo (2006) [65] | Chitosan | 0.4 | 1.2 | 2.5 | 90 | >60 cycles (>75% retention) | Polymer review | Low |
| Pillai et al. (2009) [66] | Chitosan derivatives | 0.35 | 1.1 | 2.3 | 95 | >50 cycles (>70% retention) | Solubility focus | Low |
| Rabea et al. (2003) [67] | Chitosan antimicrobial | 0.3 | 1 | 2 | 100 | >40 cycles (>65% retention) | Bio-applications | Low |
| Agnihotri et al. (2004) [68] | Chitosan nanoparticles | 0.32 | 1.05 | 2.1 | 90 | >50 cycles (>70% retention) | Drug delivery | Low |

• Quality Score:
— High: full characterization, replicate data, >100 cycles, controlled RH ±2%, error margins reported.
— Medium: basic data, limited error reporting, or <100 cycles.
—Low: incomplete methods or non-AWH-focused testing (included for contextual benchmarking, not excluded)
"Error reported" includes studies with explicit error bars, triplicate measurements, or literature-reported performance ranges with quantified variability.

**Supplementary Table S3.** Quality Scoring and Tier Assignment of Studies Included in Table S2

| Study (Author, Year) [Ref #] | Dynamic testing? | >100 cycles? | Error reported? | Realistic RH? | Degradation analyzed? | Total Score | Quality Tier | Weight |
|---|---|---|---|---|---|---|---|---|
| Kim et al. (2018) [55] | No | Yes | Yes | No | Yes | 3 | Medium | 0.6 |
| Li et al. (2023) [33] | Yes | Yes | Yes | Yes | Yes | 5 | High | 1 |

| | | | | | | | | |
|---|---|---|---|---|---|---|---|---|
| Ghaffarkhah et al. (2025) [32] | No | Yes | Yes | No | Yes | 3 | Medium | 0.6 |
| Duan et al. (2025) [70] | Yes | Yes | Yes | Yes | Yes | 5 | High | 1 |
| Shan et al. (2021) [23] | No | Yes | Yes | No | Yes | 3 | Medium | 0.6 |
| LaPotin et al. (2019) [72] | Yes | Yes | Yes | Yes | Yes | 5 | High | 1 |
| Hou et al. (2022) [48] | No | Yes | Yes | No | Yes | 3 | Medium | 0.6 |
| Fu et al. (2024) [31] | No | Yes | Yes | No | Yes | 3 | Medium | 0.6 |
| Xu et al. (2024) [40] | No | No | Yes | No | Yes | 2 | Medium | 0.6 |
| Zhou et al. (2023) [51] | Yes | Yes | Yes | Yes | Yes | 5 | High | 1 |
| Hanikel et al. (2019) [78] | No | Yes | Yes | No | Yes | 3 | Medium | 0.6 |
| Almassad et al. (2022) [86] | Yes | Yes | Yes | Yes | Yes | 5 | High | 1 |
| Yu et al. (2025) [87] | No | Yes | Yes | No | Yes | 3 | Medium | 0.6 |
| Zhao et al. (2020) [52] | Yes | Yes | Yes | Yes | Yes | 5 | High | 1 |
| Wang et al. (2014) [50] | Yes | Yes | Yes | Yes | Yes | 5 | High | 1 |
| Li et al. (2023) [44] | Yes | Yes | Yes | Yes | Yes | 5 | High | 1 |
| Goryunova et al. (2023) [39] | No | Yes | No | No | No | 1 | Low | 0.3 |
| Venkateswara Rao et al. (2006) [89] | No | Yes | No | No | No | 1 | Low | 0.3 |
| Bhagat et al. (2007) [34] | No | Yes | No | No | No | 1 | Low | 0.3 |
| Kanamori et al. (2007) [90] | No | Yes | No | No | No | 1 | Low | 0.3 |
| García-González et al. (2011) [91] | No | Yes | No | No | Yes | 2 | Medium | 0.6 |
| Kargarzadeh et al. (2018) [64] | No | Yes | No | No | Yes | 2 | Medium | 0.6 |
| Moon et al. (2011) [62] | No | Yes | No | No | Yes | 2 | Medium | 0.6 |
| Habibi et al. (2010) [63] | No | Yes | No | No | Yes | 2 | Medium | 0.6 |
| Rinaudo (2006) [65] | No | No | No | No | Yes | 1 | Low | 0.3 |
| Pillai et al. (2009) [66] | No | No | No | No | Yes | 1 | Low | 0.3 |
| Rabea et al. (2003) [67] | No | No | No | No | Yes | 1 | Low | 0.3 |

| | | | | | | | | |
|---|---|---|---|---|---|---|---|---|
| Agnihotri et al. (2004) [68] | No | No | No | No | Yes | 1 | Low | 0.3 |
| Cavka et al. (2008) [59] | No | Yes | Yes | No | Yes | 3 | Medium | 0.6 |
| Christodoulou et al. (2021) [88] | No | Yes | Yes | No | Yes | 3 | Medium | 0.6 |
| Goeminne et al. (2025) [107] | No | Yes | Yes | No | Yes | 3 | Medium | 0.6 |
| Tang et al. (2024) [105] | No | Yes | Yes | No | Yes | 3 | Medium | 0.6 |
| Ziemiański et al. (2025) [106] | No | Yes | No | No | Yes | 2 | Medium | 0.6 |
| Huang et al. (2022) [104] | No | Yes | No | No | Yes | 2 | Medium | 0.6 |
| Wang et al. (2012) [17] | No | Yes | Yes | No | Yes | 3 | Medium | 0.6 |
| Ng & Mintova (2008) [42] | No | Yes | Yes | No | No | 2 | Medium | 0.6 |
| Aristov (2009) [74] | No | Yes | Yes | No | No | 2 | Medium | 0.6 |
| Zhuravlev (2000) [73] | No | Yes | No | No | No | 1 | Low | 0.3 |
| Pekala (1989) [75] | No | Yes | No | No | No | 1 | Low | 0.3 |
| Bandosz & Ania (2006) [76] | No | Yes | Yes | No | No | 2 | Medium | 0.6 |
| Figueiredo et al. (1999) [77] | No | Yes | Yes | No | No | 2 | Medium | 0.6 |
| Du et al. (2025) [94] | Yes | Yes | Yes | Yes | Yes | 5 | High | 1 |
| Graeber et al. (2024) [41] | No | Yes | Yes | No | Yes | 3 | Medium | 0.6 |
| Yang et al. (2025) [79] | No | Yes | Yes | No | Yes | 3 | Medium | 0.6 |
| Włoch (2024) [93] | No | Yes | No | No | No | 1 | Low | 0.3 |

For quality scoring, ">100 cycles" includes studies reporting ≥80 adsorption–desorption cycles with >80% water uptake retention and evidence of material stability (e.g., post-cycling characterization). This accounts for minor variations in reporting while preserving performance relevance.
High quality (score 4–5): 9 studies → weight = 1.0
Medium quality (score 2–3): 25 studies → weight = 0.6
Low quality (score 0–1): 11 studies → weight = 0.3 (included, not excluded)

**Supplementary Table S4.** Quality Assessment Rubric (Modified Newcastle–Ottawa–Inspired Scale for Atmospheric Water Harvesting Materials)

| Quality Criterion | Met (1 pt) | Not Met / Unclear (0 pts) | Example (High = 1 pt) |
|---|---|---|---|

| | | | |
|---|---|---|---|
| Dynamic testing | Tested under flowing ambient air (e.g., 2–4 h cycles) | Static/sealed chamber only | LaPotin et al. [72] |
| Cycling stability | >100 cycles with % retention reported | <100 cycles or no retention data | Zhou et al. [51] |
| Error reporting | Replicates (n≥3) and error margins (e.g., ±) provided | No error bars or n<3 | Li et al. [33] |
| Realistic RH | Controlled ambient or salt-buffered RH (e.g., 20–40% RH) | Pure vapor or unspecified RH | Almassad et al. [86] |
| Degradation analysis | Post-cycling material characterization (e.g., SEM, XRD) | No post-stability analysis | Zhao et al. [52] |

Total score: 0–5; High quality: 4–5 pts; Medium: 2–3 pts; Low: 0–1 pt
Note: Quality scoring reflects reporting completeness and testing realism rather than intrinsic material performance.
